# Comparative Study Analysis of Epstein-Barr Virus Infection: Tissue Versus Blood Samples in Patients With Prostatic Adenocarcinoma and Its Correlation With Clinicopathological Parameters

**DOI:** 10.7759/cureus.66048

**Published:** 2024-08-02

**Authors:** Imane Mharrach, Kaoutar Anouar Tadlaoui, Abdelilah Laraqui, Khalid Ennibi, Larbi Hamedoun, Ahmed Ameur, Mohammed Alami, Anouar El Ghazzaly, Moulay Mustapha Ennaji

**Affiliations:** 1 Oncology, Faculty of Sciences and Techniques– Mohammedia, University Hassan II of Casablanca, Mohammedia, MAR; 2 Laboratory of Virology, Oncology, Biosciences, Environment and New Energies (LVO BEEN), Faculty of Sciences and Techniques– Mohammedia, University Hassan II of Casablanca, Mohammedia, MAR; 3 Center of Virology, Infectious and Tropical Diseases, Mohammed V Military Teaching Hospital, Rabat, MAR; 4 Faculty of Medicine and Pharmacy, Mohammed V University, Rabat, MAR; 5 Sequencing Unit, Laboratory of Virology, Royal School of Military Health Service, Rabat, MAR; 6 Department of Urology, Mohammed V Military Teaching Hospital, Rabat, MAR

**Keywords:** tissue, blood, biomarker, pcr, prostate cancer, epstein-barr virus

## Abstract

Prostate cancer (PCa) is the most frequently diagnosed cancer and a leading cause of cancer-related mortality in men. The diagnosis and treatment of PCa carry considerable medical, psychological, and economic implications. Among the risk factors contributing to cancer, viral infections, notably Epstein-Barr virus (EBV), play a significant role. It is recognized as an oncogenic virus associated with various lymphomas, nasopharyngeal carcinomas, and breast cancer cases but its role in PCa remains unclear. This study aims to contrast the prevalence of EBV in blood and tissue samples of PCa patients and assess its correlation with tumor clinicopathological criteria.

In this prospective study, 50 fresh biopsies and 50 blood samples were collected from patients with a confirmed diagnosis of PCa. EBV DNA was detected using polymerase chain reaction (PCR). A statistical analysis was then conducted to examine the correlation between EBV prevalence and PCa clinicopathological characteristics. EBV DNA was detected in 38% of PCa blood samples and 64% of PCa tissue samples, with a higher prevalence in tissue samples (p = 0.009). The statistical analysis revealed a significant correlation between EBV infection and pathological Gleason score (p = 0.041) in PCa tissue, as well as pathological T-stage (p = 0.02) in PCa blood.

The results show that patients with PCa have higher levels of EBV in their tissues than in their blood, suggesting that EBV may play an important role in the etiology of PCa. This paves the way for further research into the function of EBV as a potential biomarker in the development and progression of prostate carcinoma in order to combat oncogenic viruses.

## Introduction

Prostate cancer (PCa) is the most prevalent cancer among men and ranks as the fourth most commonly diagnosed cancer worldwide. According to GLOBOCAN (Global Cancer Observatory) 2020, there were reportedly 1,414,259 new PCa cases, accounting for 7.3% of all cancer cases. Additionally, PCa resulted in approximately 375,304 deaths, representing 3.8% of all cancer-related deaths, making it the fifth leading cause of cancer mortality globally [1]. Many factors contribute to the development of PCa, including family history, age, and race/ethnicity. In addition, lifestyle habits such as alcohol consumption and smoking play a significant role [2]. On the other side, many studies are directing their focus toward examining the impact of viral infections on the development of PCa. Oncogenic viruses contribute to approximately 12% of cancers worldwide [3]. These infections can function as indirect carcinogens, where the infection results in carcinogenic mutations, or as direct carcinogens, when viral oncogenes directly contribute to the transformation of cancer cells [4]. PCa is commonly linked to several viruses, including human papillomavirus (HPV), human herpesvirus 8 (HHV8), Epstein-Barr virus (EBV), and human herpes simplex virus 2 (HHV2) [5,6].

EBV, also known as human herpesvirus 4, is a lymphotropic herpesvirus responsible for infectious mononucleosis. It has a 172 kbp double-stranded DNA (dsDNA) genome [7]. Similar to other members of the *Herpesviridae* family of viruses, EBV can become latent in the cells of the infected organism and can occasionally reactivate in the lytic phase [8]. It causes a persistent infection in memory B lymphocytes, characterized by the presence of latent EBV infection markers such as EBV core antigen 1 and latent membrane protein 2 [9]. During latency, EBV can reactivate and stimulate viral genes, including EBNAs (Epstein-Barr nuclear antigens), *LMP1,* and *LMP2*. This reactivation promotes epithelial cell growth, proliferation, and angiogenesis while also inhibiting apoptosis [10].

The oral route is the principal mode of transmission for EBV. However, organ transplants and blood transfusions are other potential modes of EBV transmission. EBV infection is commonly found in the population and has been related to an increasing number of epithelial and lymphocytic carcinomas, including gastric adenocarcinoma, nasopharyngeal carcinoma, T-cell lymphoma, Burkitt's lymphoma, and Hodgkin's lymphoma [11,12]. Some researchers have suggested that epithelial carcinomas, such as those of the breast, colon, lung, and prostate, may also be included in the list of EBV-related tumors [13]. Other research has been carried out to study the potential association between EBV and the development of PCa [14-16]. This study seeks to conduct a comparative analysis of EBV infection in patients diagnosed with prostatic adenocarcinoma, focusing on the examination of both tissue and blood samples and finding correlations between this viral infection and tumor clinical criteria. This comparative analysis aims to advance our understanding of EBV infection in prostatic adenocarcinoma. The findings of this study have the potential to inform clinical practice by identifying novel diagnostic biomarkers and therapeutic targets for improved management of PCa.

## Materials and methods

Sample selection and characteristics

In this study, a total of 100 samples of 50 PCa patients and 50 controls were collected from the Teaching Hospital of Rabat City, Morocco, between April 2023 and March 2024. Histological samples were obtained and examined for prostate adenocarcinomas. Informed permission was obtained from all participants collaborating in the study. The Committee of Biomedical Research Ethics in Morocco approved the study (3/2018/30 April/2018).

Fresh prostate biopsies and blood samples were obtained according to standard protocols by physicians directly. Every sample had the relevant attached clinical and pathological parameters, including age, date of diagnosis, date of biopsy, Gleason score, prostate-specific antigen (PSA) concentration of prostate tumors, and the vital status of the patients. Exclusion criteria for the study were patients who received chemotherapy or radiotherapy treatments.

The clinical characteristics of PCa patients are provided in Table 1.

**Table 1:** Clinical characteristics of prostate cancer patients (N=50) PSA: prostate-specific antigen

Characteristics	n (%)
Age at Diagnosis/Surgery	
< 60 Years	13 (26%)
⩾ 60 Years	37 (74%)
PSA (ng/ml)	
< 2.5	2 (4%)
2.5 – 10	12 (24%)
⩾ 10	36 (72%)
Pathological Gleason Score	
⩽6	14 (28%)
> 6	36 (72%)
Pathological T-Stage	
T1	17 (34%)
T2 X	29 (58%)
T3 X	1 (2%)
T4	3 (6%)
Alcohol Consumption	
Yes	28 (56%)
No	12 (24%)
Weaned	10 (20%)
Smoking	
Yes	32 (64%)
No	11 (22%)
Weaned	7 (14%)

Viral DNA extraction

Total DNA was extracted from tissues and blood samples using the QIAamp DNA Mini Kit (QIAGEN N.V., Hilden, Germany) according to the kit protocol for PCa tissues and blood. DNA was quantified using a Nanodrop spectrophotometer (Thermo Fisher Scientific Inc., Waltham, Massachusetts, United States). Polymerase chain reaction (PCR) was conducted on samples with a DNA concentration of 20-50 ng/µl or higher. The quality of the extracted DNA was analyzed by amplifying a 268 bp fragment of the β-globin gene using HotStarTaq DNA polymerase (QIAGEN N.V.) in the presence of GH20/PCO4 primers (Table 2). The cycling program consisted of nine minutes at 95°C followed by 35 cycles of an extension cycle of 30 seconds at 95°C, 30 seconds at 55°C, one minute at 72°C, and finally 10 minutes at 72°C.

**Table 2:** The primer sequences used for PCR tests PCR: polymerase chain reaction

Oligonucleotide	Sequences of primers	Tm	PCR Product Sizes
B-globin	PC04 5'-CAA CTT CAT CCA CGT TCA CC-3'	54°C	256 bp
	GH20 5'-GAA GAG CCA AGG ACA GGT AC-3'		
EBV	EBNA-2 OP – F 5'-GCG GGT GGA GGG AAA GG-3'	58°C	168 pb
	EBNA-2 OP – R 5'-GTC AGC CAA GGG ACG CG-3'		
	EBNA-2 IP – F 5'-AGG CTG CCC ACC CTG AGG AT-3'	66°C	
	EBNA-2 IP – R 5'-GCC ACC TGG CAG CCC TAA AG-3'		

Amplification of EBV by nested PCR

All positive β-globin gene PCR products were further confirmed by PCR. To be sure of the reliability of the assays, a positive control was performed during handling.Specific EBV genes were targeted by PCR using specific primers (Table 2), and amplification of EBV DNA was performed using a nested PCR with two specific primers’ pairs.

The PCR reaction consisted of a PCR reaction with a total volume of 25 µl, genomic DNA (8 ng), 2x Taq PCR Master Mix kit (QIAGEN N.V.), and 2 µmol forward and reverse primers. PCR amplification using a thermal cycler amplification procedure for EBV gene amplification processing was as follows: initial denaturation at 94°C for three minutes, followed by denaturation at 94°C for one minute, annealing at 43°C for one minute, extension at 72°C for one minute, and a final extension of 35 cycles at 72˚C for 10 minutes. The PCR products were resolved using 2% agarose gel electrophoresis for 1.5 hours at 70 V, then stained with 1% ethidium bromide and visualized under UV light.

Statistical analysis

Results were analyzed using SPSS 11.0 for Windows Student Version (Released 2001; SPSS Inc., Chicago, United States). Data were calculated on 2x2 or more Chi‑squared analysis or Fisher's exact test to assess the significance of the association between clinicopathological data (patient’s age, histological grade, and tumor stage) in correlation with the presence of EBV. Statistical significance was achieved at p < 0.05.

## Results

**Figure 1:**
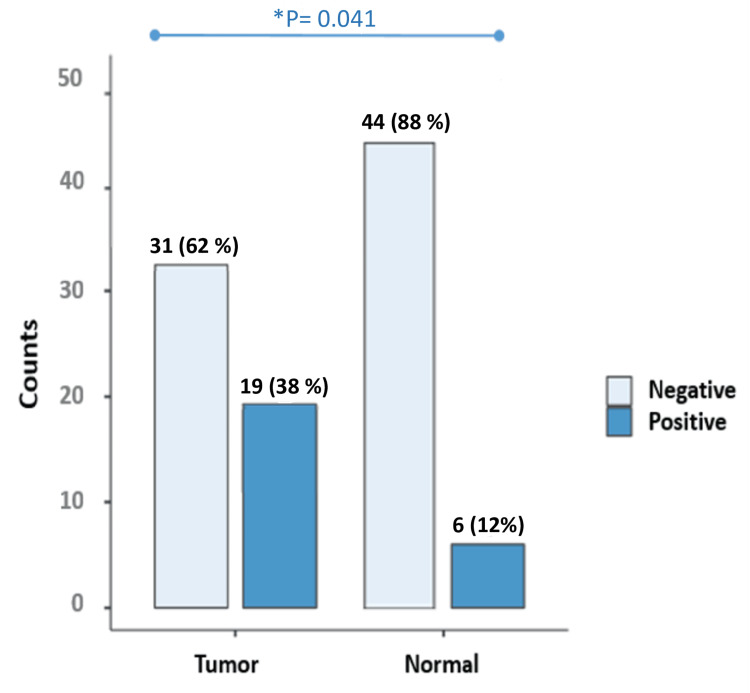
EBV status in blood from the patients with prostate cancer (Tumor) vs. that from the non-cancer control group (Normal) *p value < 0.05 is considered significant EBV: Epstein-Barr virus

**Figure 2:**
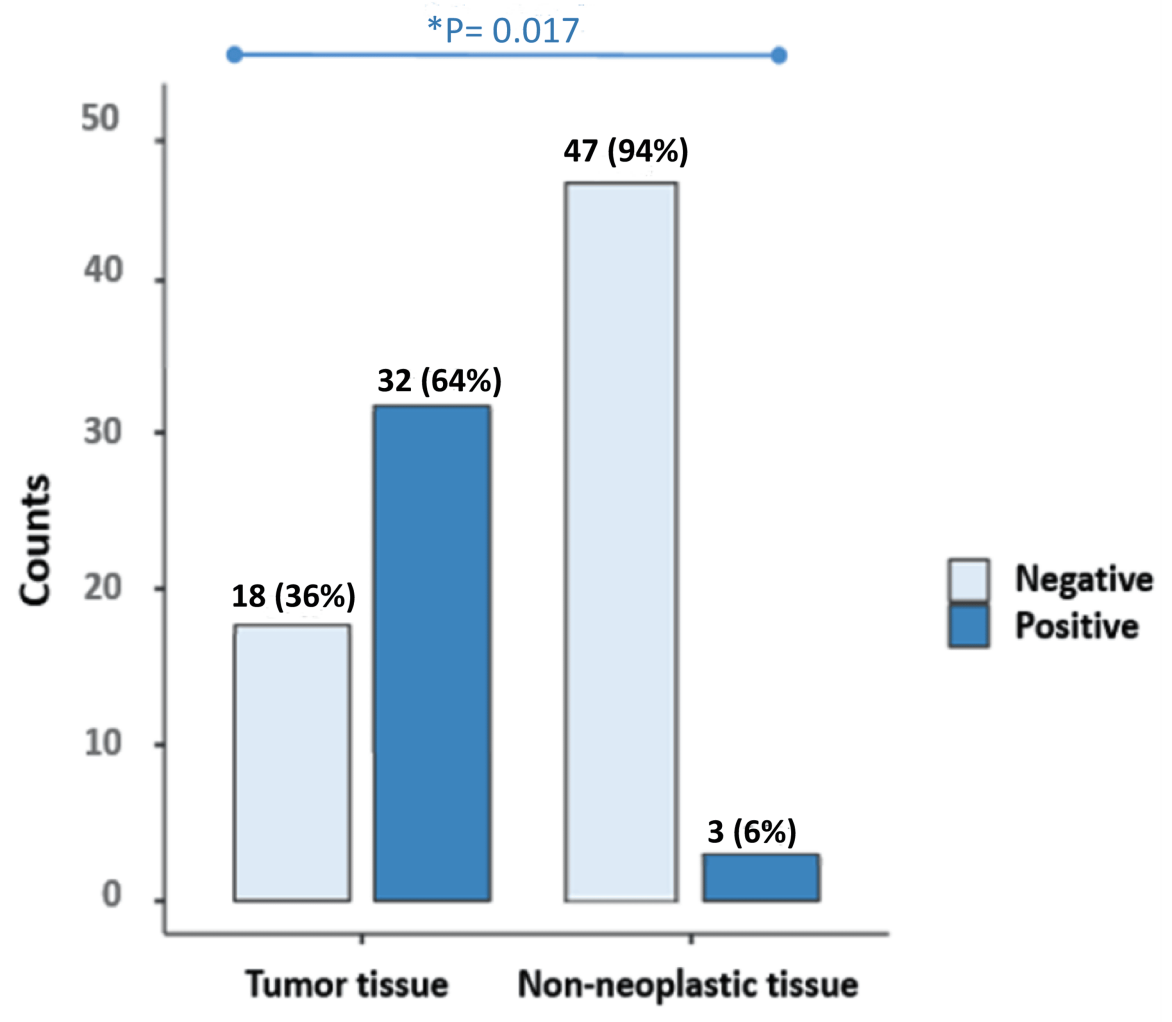
EBV status in cancerous prostate tissue (Tumor) versus non-neoplastic prostate tissue *p-value < 0.05 is considered significant EBV: Epstein-Barr virus

Initially, DNA quality was examined after the extraction process by performing a PCR reaction targeting a 256-bp sequence in the β-globin gene. Successful amplification of this gene in all samples indicated the presence of high-quality DNA suitable for our study. Subsequently, PCa samples were analyzed for the presence of EBV, revealing EBV DNA in 64% of tissue samples. Similarly, EBV DNA was detected in 38% of blood samples from PCa patients. In tissue and blood controls, 6% and 12% of EBV DNA were positive, respectively. The prevalence of EBV infection was significantly higher in tissue samples compared to blood samples (p = 0.009). EBV was found to be significantly more prevalent in tissue and blood samples from men with PCa than in samples from cancer-free controls (p < 0.05) (Figure 1, Figure 2 ).

Based on Table 3, for tissue samples that are EBV positive, the mean age at diagnosis is 68 years, indicating that the sample consists of older individuals, with ages ranging from 55 to 82 years. The majority of patients were aged over 60 years, comprising 81% of the total cohort. There was no significant association between age at diagnosis/surgery and the presence of EBV (p = 0.659). Regarding PSA levels, a significant proportion of patients (72%) exhibited elevated PSA levels (≥10 ng/ml), which can be a marker for the presence of PCa with limitations in terms of specificity, the results do not reveal any relationship between PSA and the presence of EBV (p=0.524). Pathological Gleason scores varied within the cohort, with approximately more than half of the patients (78%) presenting with scores above 6, while a smaller subset had scores below 6 (22%). There is a significant association between higher Gleason scores and the presence of EBV (p = 0.041). Patients with higher Gleason scores (>6) are more likely to have EBV-positive tumors compared to those with lower scores. Analysis of pathological T-stages showed a predominant distribution in the T2 stage (59%) and T1 stage (35%); there was no significant association between T-stages and the presence of EBV (p = 0.652). Lifestyle factors also had an impact, with a notable proportion of patients reporting alcohol consumption (72%) and smoking habits (53%) with a significant association between alcohol consumption and smoking status and the presence of EBV, respectively (p = 0.001; p = 0.036).

**Table 3:** Association between viral infection and clinicopathological characteristics for tissue and blood * p ⩽ 0.05 is a significative value PSA: prostate-specific antigen

Tumor ﻿characteristics	Number of cases tested	EBV in tissue (n=32/50), n (%)	P-value	EBV in blood (n = 19/50), n (%)	P-value
Age at Diagnosis					
< 60 Years	13 (26%)	6 (19%)	0.659	5 (26%)	0.968
⩾ 60 Years	37 (74%)	26 (81%)		14 (74%)	
PSA (ng/ml)					
< 2.5	2 (4%)	2 (6%)	0.524	1 (5%)	0.566
2.5 – 10	12 (24 %)	7 (22%)		3 (16 %)	
⩾ 10	36 (72 %)	23 (72%)		15 (79 %)	
Pathological Gleason Score					
⩽ 6	14 (28%)	7 (22%)	0.041*	7 (37%)	0.276
> 6	36 (72%)	25 (78%)		12 (63 %)	
Pathological T-Stage					
T1	17 (34%)	11 (35%)	0.652	5 (26%)	0.02*
T2 X	29 (58%)	19 (59%)		11 (58%)	
T3 X	1 (2%)	1 (3%)		1 (5%)	
T4	3 (6%)	1 (3%)		2 (10 %)	
Alcohol Consumption					
Yes	28 (56 %)	23 (72%)	0.001*	9 (47%)	0.303
No	12 (24%)	7 (22 %)		4 (21%)	
Weaned	10 (20 %)	2 (6%)		6 (32%)	
Smoking					
Yes	32 (64%)	17 (53%)	0.036*	13 (69%)	0.246
No	11 (22%)	8 (25%)		2 (10%)	
Weaned	7 (14%)	7 (22%)		4 (21%)	

For blood samples from PCa patients infected with EBV, 81% of these patients were over the age of 60. Regarding PSA levels, a significant proportion (79%) exhibited elevated PSA levels (≥10 ng/ml). However, there was no significant association between PSA levels and the presence of EBV (p = 0.524). Among the 19 EBV-positive patients, 63% had a Gleason Score greater than 6. However, there was no statistically significant association between pathological Gleason scores and the presence of EBV (p = 0.276). Regarding tumor stage, 58% of patients had T-stage 2 tumors, and 26% had T-stage 1 tumors. There was a significant difference between T-stage and EBV infection (p = 0.02). It was found that EBV DNA was present in 47% of patients who consumed alcohol and 69% of those who smoked. However, the associations with alcohol consumption and smoking were not statistically significant (P=0.303; P=0.246).

## Discussion

PCa is the second-most common cancer among men globally [17]. Research indicates that approximately 12% of cancers worldwide are attributed to viral infections [18]. Several studies have indicated a correlation between PCa and specific viral infections, such as HPV, EBV, BK virus, and SV4 [19-22]. However, in Moroccan men, the detection and characterization of EBV in PCa tissues compared to blood samples, along with its correlation with clinicopathological parameters, have not been previously studied. Our study aimed to compare EBV infection rates between tissue and blood samples obtained from patients with prostatic adenocarcinoma and evaluate its association with clinicopathological parameters.

We found a notable difference in EBV detection rates between tissue and blood samples. While tissue samples exhibited a higher prevalence of EBV infection (64%), blood samples also showed considerable rates of EBV positivity (38%). However, in the control group, EBV DNA was positive in 6% and 12% of the tissue and blood samples, respectively. This discrepancy underscores the importance of utilizing multiple sample types to comprehensively assess EBV infection in PCa patients. On the other hand, the frequency of EBV infection was notably elevated in tissue samples in comparison to blood samples, demonstrating a statistically significant distinction (p = 0.009). EBV was significantly more prevalent in both tissue and blood samples from men with PCa compared to samples from non-cancer controls (p < 0.05), as supported by the study conducted by Sfanos et al. [23] (P = 0.019). This indicates that EBV infection is more frequently detected in PCa cases, suggesting a potential association between EBV and the development or progression of PCa [24].EBV was more prevalent in blood samples than in tissue samples among non-cancer controls. 12% of blood samples from individuals without cancer (non-cancer controls) show detectable EBV DNA, whereas only about 6% of tissue samples from the same group exhibit the presence of EBV.

Several research groups from Poland and Iran have noted high levels of EBV detection in PCa tissue. Jacek et al. [24] and Nahand et al. [19] identified EBV in 49% and 49.3%, respectively. In another study by Grinstein et al. in the United States, approximately 37% of PCa patients were found to have EBV infection using the immunohistochemistry method [25]. Our findings are generally similar to those discussed previously. However, a study conducted in Sweden by Bergh et al. found that the prevalence of EBV infection in prostate tissues was 9.4% among PCa patients and 8.8% in the control group [26]. In another study in the United States, EBV was found in 8% (16 out of 200) of all normal, benign, and malignant prostate tissues [23]. These findings suggest that EBV may not have a significant role in the development of PCa. However, the impact of EBV on the initiation and development of PCa remains unclear.

The presence of EBV infection in tissue demonstrated a significant association with tumor criteria, particularly showing differences in Gleason score, alcohol consumption, and smoking habits, with respective p-values of 0.041, 0.001, and 0. 036. The majority of infected men's tissue (25 out of 32 patients) exhibited a Gleason score exceeding 6, indicating a possible link between EBV and higher-grade of PCa. Other authors have reported similar findings when comparing histopathological parameters based on the Gleason score [16,24]. Furthermore, 94% of these patients displayed tumor pathological stages at either stage one or two (T1 35%, T2 59%), underscoring the early developmental stage of their prostate tumors. This suggests a potential association between viral infections and the initiation of PCa, with viral presence observed early in the tumorigenesis process. In a research we did in our laboratory, 72% of individuals with PCa had EBV in their blood [27], The variation in reported percentages of EBV positivity in PCa blood samples highlights the complexity of studying viral presence in cancer. Differences in sample populations, detection methods, and study methodologies can contribute to discrepancies in findings. When discussing such discrepancies, it's important to consider factors such as sample size, patient demographics, stage of cancer, and the sensitivity of the detection assay used. Additionally, variations in the techniques used for EBV detection, such as PCR-based assays or serological methods, can also impact the reported percentages.

﻿The association between EBV infection in blood and tumor criteria indicated a significant difference specifically in the pathological T-stage (P=0.02). This finding is similar to other studies [27]. These findings led us to conclude that EBV viral infections were present during the early stages of tumor development. Based on the analysis of both blood and tissue samples, the findings suggest that EBV infections are implicated in the early stages of tumor development and initiation of PCa. Further research is necessary to elucidate the exact mechanisms through which EBV influences tumor initiation and progression, which could potentially lead to new diagnostic and therapeutic strategies targeting viral infections in PCa. Infections with EBV can vary in frequency across different geographical regions [28]. Discrepancies in study outcomes may also be influenced by factors such as the limited size of the study population, the type of clinical samples used, and the method of virus detection employed. Given that EBV is known to infect epithelial cells and can establish latency, its presence in prostate tissue might facilitate chronic inflammation or directly promote oncogenesis through its viral proteins.

The higher prevalence of EBV in prostate tissue compared to blood (p = 0.009) suggests that EBV may preferentially localize or replicate in prostate tissue. This finding supports the hypothesis that EBV could play a role in prostate tumor biology, possibly through localized infection and subsequent oncogenic effects. EBV may contribute to PCa development by modulating the tumor microenvironment, promoting inflammatory responses, or through direct oncogenic effects via viral proteins such as LMP1 and EBNA1.

This study is limited by its cross-sectional design and relatively small sample size. Further studies with larger cohorts and longitudinal follow-up are needed to verify the previous hypothesis, investigate the mechanistic role of EBV in PCa, and assess the potential of EBV as a biomarker for diagnosis or as a therapeutic target.

## Conclusions

﻿The involvement of EBV infection in the development of PCa is a topic that has received little attention from researchers. However, EBV may contribute to the development and/or progression of PCa. Our results support the hypothesis that EBV may preferentially localize to or be more actively involved in prostate tissue, potentially contributing to tumor development and progression. Further research is needed to understand the mechanisms underlying this association and its clinical implications.
